# Editorial: Marching Toward 100% Whole Genome Sequencing

**DOI:** 10.3389/fgene.2016.00041

**Published:** 2016-03-31

**Authors:** Yih-Horng Shiao

**Affiliations:** Department of Commerce, US Patent Trademark OfficeAlexandria, VA, USA

**Keywords:** dynamic genome, repetitive sequences, genome sequencing, mapping, assembly, diagnosis

Genomes are becoming recognized as dynamic molecules which modify their structures, mediated through epigenetic mechanisms, in response to intrinsic and extrinsic stimulants, and directed by cell development in single-cell organisms and mammalian cells, examples including V(D)J recombination during B-cell and T-cell lymphocyte maturation, and subsequent class switch recombination and somatic hypermutation in the constant segment of the IgH locus to enhance antibody affinity (Shiao, 2015). Ontogeny-driven rearrangement, methylation, and transcription in mouse tandemly repeated 45S *rDNA* suggests that dynamic genome restructuring plays a key role in embryonic development and tissue differentiation in mammals (Shiao et al., 2011). Any mistakes accompanying this genome restructuring process can introduce mutations, examples including oncogenic fusions to immunoglobulin, T-cell receptor, and 28S *rDNA* genes (Kobayashi et al., 2014), and the Robertsonian translocation known to increase risk of miscarriage, infertility, and congenital abnormalities (Alfarawati et al., 2012). Whole genome sequencing becomes a critical tool to determine the extent of restructuring and to assist in deciphering the mechanisms of genome rearrangements.

In this Research Topic, a collection of eight articles touches upon various aspects of whole genome sequencing, encompassing challenges to obtain 100% genome sequence coverage or reliable reference genomes, mapping and assembly of short-read sequencing data, detection of copy number variation across large genome segments, progress in long-read single molecule sequencing technologies, and clinical applications of exome and genome sequencing. One major challenge is associated with sequence reads that are insufficiently long to span long repetitive sequences, which are interspersed in the genome or can be concentrated in telomere, centromere, and acrocentric regions. These articles provide some directions that should bring whole genome sequencing closer to the goal of 100% completion.

Mascher and Stein recommend a workflow for creating a genetically anchored whole-genome shot gun (WGS) assembly: (i) construction of a WGS assembly from next-generation sequencing (NGS) data in individual genomes, (ii) mapping of the sequence reads of a segregating population to the assembly to allow detection of single-nucleotide polymorphisms (SNPs), (iii) building a genetic linkage map, and (iv) integration of the contigs harboring the WGS SNPs to the linkage map. The workflow is particularly useful for non-model but economically important species, for which high-quality reference genomes are still under construction.

Li and Freudenberg employ a computational model to demonstrate that the read length of genome sequencing needs to be increased exponentially to convert unmappable regions/reads to mappable, especially in highly repetitive genome regions stretching across 10,000 or more bases, such as telomeres, centromeres, short-arms of acrocentric chromosomes, and large heterochromatic regions.

Jiang et al. present an approach combining conventional bacterial artificial chromosome (BAC) end sequences with physical map contig-specific sequences (PMCSS) to increase the total length of genome contigs for scaffolding. PMCSSs are obtained by restriction digestion with two enzymes, followed by tagging of digested fragments with barcoded adaptors, amplification, sequencing, and decoding of each fragment for assembly. The approach is valuable for assembly of repetitive sequences and/or complex genomes which go through multiple rounds of genome duplication.

Peters et al. propose a strategy for achieving an error-free “perfect genome” from inputs of 10 to 100 cells, by distribution of fragmented long DNAs into individual nano-compartments, followed by on-site fragmentation and tagging with compartment-specific barcoded sequences, pooling of tagged DNAs, pair-end massively parallel sequencing of about 300 bases, and unbiased phased de novo assembly of sequences. The strategy is capable of resolving paternal/maternal alleles and repetitive sequences, and is applicable to analyses of *in vitro* fertilized embryo biopsies, circulating tumor cells, and circulating fetal cells.

Glusman et al. describe a method to identify copy number variants (CNVs) of 1–100 kb in individual genomes, based on a comparison to Reference Coverage Profiles (RCP) pre-computed from over 6000 high quality (>40X) genomes. Their strategy is to compress raw genome coverage data from short-read sequencing hundreds-fold, followed by scaling of the genome coverage stratified at 1 kb-resolution into 25-type %GC ranges to the total autosomal coverage and further to average coverage from a set of genomes obtained by specific technology. Next, 1-kb resolution RCF represented by median scaled coverage is generated to serve as an estimator of diploid genome, followed by normalization of individual scaled coverage in each kb-sized region to the corresponding RCF value to obtain a normalized coverage profile (NCP). Finally, segmentation of the NCP is performed using hidden Markov model (HMMSeg) for ploidy calling, followed by computation of CNVs frequencies.

Wang et al. overview progress in protein and solid-state nanopore sequencing technologies, and offer many tips for improving such technologies. The areas to be improved include detection of ionic current blockage, reduction of signal interference from neighboring bases, discrimination of electronic tunneling signal by an electrode which can be further modified to enhance its interaction with bases, and transistor-based sequencing devices.

Alvarez et al. introduce a statistical method to account for correlations between currents to reduce call error of transverse current-based DNA/RNA nanopore sequencing. The error comes from intrinsic noise in the current arising from interaction of molecular orbitals of neighboring bases along the chain of the DNA or RNA molecule. They also demonstrate that aluminum and graphene electrodes produce near 100% fidelity of base calling in 200-bp random sequences, human insulin and BRCA1 genes, and homopolymers after applying the statistical method.

Shen et al. review clinical applications of massively parallel next-generation sequencing of exomes and/or genomes in cancer diagnosis and treatment. They provide examples of clinically relevant somatic mutations and structural rearrangements in several cancer types and compile a list of commercial laboratories which offer such exome- or genome-based sequencing service for clinical uses. These clinical applications will surely assist in development of personalized or precision medicine.

Recently, reports of read length of over 50 kb using a nanopore sequencer in conjunction with short-read sequencing provide proof of the principle that nanopore sequencing has the potential to directly sequence entire regions containing long repetitive sequences (Goodwin et al., 2015; Madoui et al., 2015). The high error rate of the nanopore technology still represents a bottleneck for extending the read length.

In sum, major progress in marching toward 100% genome sequencing has been made, but many challenges remain. These challenges include routinely obtaining long reads (preferably 100 kb or more) with high fidelity to encompass large repetitive sequences and CNVs, being able to handle the low quantities of native DNA/RNA typically employed for clinical or cell-specific diagnoses, and developing robust algorithms to assist in sequence call, genome assembly, and clinical interpretation from the torrents of sequence data that are increasingly becoming available. Novel technologies, strategies, and ingenuity are needed for further improvement of whole genome sequencing.

## Author contributions

The author confirms being the sole contributor of this work and approved it for publication.

### Conflict of interest statement

The author declares that the research was conducted in the absence of any commercial or financial relationships that could be construed as a potential conflict of interest. The views expressed do not necessarily represent those of the US Federal agency.

## References

[B1] Alfarawati S. Fragouli E. Colls P. Wells D. (2012). Embryos of Robertsonian translocation carriers exhibit a mitotic interchromosomal effect that enhances genetic instability during early development. PLoS Genet. 8:e1003025. 10.1371/journal.pgen.1003025 23133396 PMC3486902

[B2] Goodwin S. Gurtowski J. Ethe-Sayers S. Deshpande P. Schatz M. C. McCombie W. R. (2015). Oxford Nanopore sequencing, hybrid error correction, and de novo assembly of a eukaryotic genome. Genome Res. 25, 1750–1756. 10.1101/gr.191395.115 26447147 PMC4617970

[B3] Kobayashi S. Taki T. Nagoshi H. Chinen Y. Yokokawa Y. Kanegane H.. (2014). Identification of novel fusion genes with 28S ribosomal DNA in hematologic malignancies. Int. J. Oncol. 44, 1193–1198. 10.3892/ijo.2014.2291 24504345

[B4] Madoui M.-A. Engelen S. Cruaud C. Belser C. Bertrand L. Alberti A.. (2015). Genome assembly using Nanopore-guided long and error-free DNA reads. BMC Genomics 16:327. 10.1186/s12864-015-1519-z 25927464 PMC4460631

[B5] Shiao Y.-H. (2015). Interplay of epigenetics, genome rearrangement, and environment during development, in Environmental Epigenetics, Molecular and Integrative Toxicology, eds Su L. J. Chiang T.-C. (London: Springer-Verlag), 281–294.

[B6] Shiao Y.-H. Leighty R. M. Wang C. Ge X. Crawford E. B. Spurrier J. M.. (2011). Ontogeny-driven rDNA rearrangement, methylation, and transcription, and paternal influence. PLoS ONE 6:e22266. 10.1371/journal.pone.0022266 21765958 PMC3134480

